# Clock-modified mesenchymal stromal cells therapy rescues molecular circadian oscillation and age-related bone loss via miR142-3p/Bmal1/YAP signaling axis

**DOI:** 10.1038/s41420-022-00908-7

**Published:** 2022-03-12

**Authors:** Sa Cha, Jiangyue Wang, Sueng Min Lee, Zhen Tan, Qing Zhao, Ding Bai

**Affiliations:** State Key Laboratory of Oral Diseases & National Clinical Research Center for Oral Diseases, West China Hospital of Stomatology, Sichuan University, Chengdu, China

**Keywords:** Ageing, Bone remodelling

## Abstract

Age-related bone loss and disease strongly affect the quality of life of the elderly population. Cellular circadian rhythms have been reported to regulate bone aging, and micro RNAs (miRNAs) play crucial posttranscriptional regulatory roles in the peripheral clock network. Proliferation capability, osteogenic lineage commitment, senescence-associated secreted phenotype (SASP) and circadian oscillation of clock genes under osteogenic condition were assessed in bone marrow mesenchymal stromal cells (BMSCs) from young adult and aged adult mice. miRNAs targeting the core clock gene brain and muscle arntl-like protein 1 (Bmal1) were screened and verified in young and old BMSCs with RT-qPCR and Western Blot analysis. ChIP-seq and RNA-seq datasets were mined to define the downstream mechanism and gain- and loss-of-function genetic experiments were performed to confirm the hypothesis. To compare the therapeutic effect of these clock-engineered BMSCs, SASP and osteogenic capability of Bmal1-overexpressing and miR-142-3p-inhibited BMSCs were investigated in vitro and transplanted into bone defects and femur cavities of aged mice. Aged BMSCs displayed an abolished circadian rhythm, impaired self-renewal capability and decreased osteoblast differentiation. miR-142-3p was elevated with aging, which downregulated Bmal1 and diminished the osteogenic potential of BMSCs. In addition, Bmal1 inhibited YAP expression to promote BMSCs osteogenesis, which was independent from the activation of Hippo signaling pathway. Overexpression of Bmal1 or inhibition of miR-142-3p rescued the molecular temporal rhythm and osteoblast differentiation ex vivo. Cell-based circadian therapy showed improved bone formation and higher turnover levels in vivo. This study demonstrates that transcriptional and post-transcriptional level clock-modified BMSCs rescued circadian oscillation and age-related bone loss via miR-142-3p/Bmal1/YAP signaling axis. These data provide promising clinical prospects of circadian-mediated stromal cell-based therapy and bone tissue regeneration.

## Introduction

Age-related bone loss and defects affect millions of individuals and generate a large economic burden, which features a decrease in bone mass and impaired repair capability [1, 2]. Clinical interventions to prevent these deleterious conditions have been proposed, including MSCs therapy, which spreads multiple differential progenitors to defects and recruits endogenous MSCs for tissue regeneration [3]. Bone marrow mesenchymal stromal cells (BMSCs) constitute a specific portion of adult stem cells within the bone marrow that can differentiate into osteoblasts in response to external stimuli and are necessary for bone tissue regeneration and remodeling. Age-associated intrinsic changes in BMSCs include loss of stemness, cell senescence and defective osteoblast differentiation, which leads to the gradual exhaustion of their regeneration potential and impairs bone integrity [4–6]. These age-dependent traits limit the usage of BMSCs bone tissue engineering [7]. Therefore, additional research is urgently needed to obtain a better treatment effect in bone tissue regeneration.

Cellular molecular oscillations are regulated by BMAL1, and CLOCK comprises heterodimers that drive the daily changes in the transcription of circadian controlled genes (CCGs) [8–11]. Mutation of the core clock gene Bmal1 results in disorganized circadian oscillations and prematurity with a loss of bone mass in mice [12, 13]. Our previous study showed that BMAL1 expression decreased in 16-month MSCs along with a loss of differentiation capability [14, 15], but the molecular basis and bone-specific circadian alterations in aging are less understood. Micro RNAs (miRNAs) are endogenous small noncoding RNAs that regulate gene expression and orchestrate physiological activities at the posttranscriptional level. Several studies have reported the cell type-specific regulation of miRNAs in circadian rhythms [16–18]. As endogenous small RNAs, miRNAs can be chemically modified and easily delivered to the target site, making them interesting candidates for therapeutics [19]. The tissue-specific clock orchestrates divergent organ functions and has been primarily adopted, but how the molecular clock system coordinates peripheral circadian functions in aging remains unknown [20].

Hippo pathway was one of the crucial signaling networks regulating tissue homeostasis and development. YAP worked as the terminal transcriptional activator of Hippo pathway, co-regulated with TAZ, and affected target genes’ expression. Controversial evidence has been reported on the function of YAP in bone homeostasis in vitro and in vivo. The symphony of this co-transcriptional factor is still not fully understood. Recent studies showed that YAP1/NF-κB suppress clock-mediated unfolded protein responses and promotes proliferation in sarcoma, highlighting the potential mediated role of Hippo pathway peripheral circadian oscillation and stem cells related cellular functions. The study aimed to explore Bmal1-mediated peripheral circadian rhythm changes and underlying mechanisms in aged BMSCs, to develop a pilot concept for clock-engineered cell-based therapy in bone tissue regeneration.

## Results

### Circadian molecular rhythm alterations, stemness decreases and cell senescence with aging in BMSCs

**Fig. 1:**
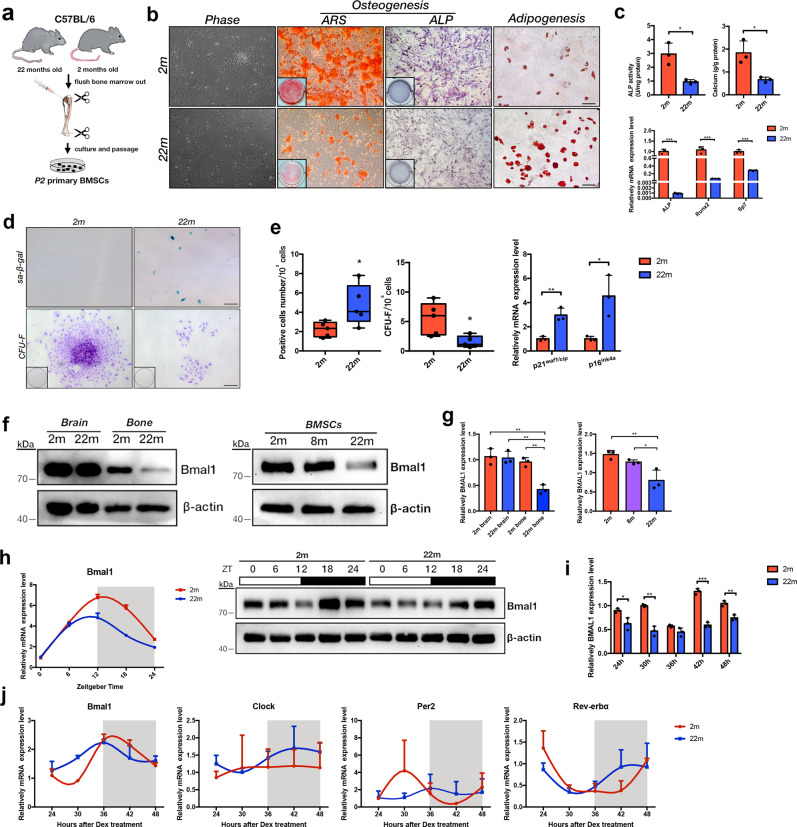
The circadian molecular rhythm was abolished, and Bmal1 expression decreased in bone and BMSCs with aging. **a** Workflow diagram of BMSCs isolation. **b** Morphological images, Alizarin red, ALP and Oil Red staining of 2-months and 22-months BMSCs under osteogenic and adipogenic conditions. Scale bar: 200 μm. **c** Quantitative analysis of ALP and ARS staining and qRT-PCR analysis of osteogenic-related gene expression (*Alp, Run2*, and *Sp7*) in 2-month and 22-month BMSCs. **d** CFU-F and SA-β-gal staining of 2-months and 22-months BMSCs. Scale bar: 200 μm. **e** Quantitative analysis of the numbers of colonies and senescent cells, qRT-PCR analysis of senescence-associated secreted phenotypes (*p16*^*ink4a*^*, p21* ^*waf1/cip*^*)*. **f** Western blot analysis of *Bmal1* expression in Brain tissue, Bone tissue and BMSCs in mice with different ages. **g** Quantitative analysis of data in f. **h** qRT-PCR and western blot analysis of *Bmal1* circadian expression in BMSCs aged two months and 22 months. Cells were harvested at ZT0, ZT6, ZT12, ZT18, and ZT24. **i** Quantitative analysis of data in (**h**). **j** qRT-PCR analysis of molecular rhythmic expression of circadian clock genes (*Bmal1, Clock, Per2*, and *Rev-erbα*) in 2-month and 22-month BMSCs under osteogenic conditions. *N* = 3-5, Data are presented as mean ± SD; *p < 0.05, **p < 0.01, ***p < 0.001 (**c**, **e**, **i**) by two-tailed Student’s *t*-tests and (**g**) by one-way ANOVA with Tukey’s post hoc test.

Primary BMSCs were isolated from the femur and tibia of 2-month-old and 22-month-old mice as previously described [14] (Fig. 1a). Representative images of multilineage differentiation assay (ARS, ALP and oil red staining) showed that the osteogenic and adipogenic capability of BMSCs decreased with aging. The expression of osteogenic-related markers was significantly reduced at the transcriptional level on 22-month compared with 2-month (Fig. 1b, c). Colony formation- and senescence-positive cells were assessed by CFU-F and SA-β-gal staining in the two age groups. The aged BMSCs exhibited limited self-renewal capability and increased numbers of senescent cells compared with the young BMSCs (Fig. 1d, e). Consistent with the SA-β-gal staining, p16^ink4a^ and p21^waf1/cip^ mRNA expressions were elevated in aged cells (Fig. 1e). To compare the age-related BMAL1 circadian expression in the central and peripheral tissue, brain and bone tissue were harvested from young and old mice. There was a significant decrease in BMAL1 expression in cranial bone. However, BMAL1 expression in brain was relatively consistent with age (Fig. 1f, g). To further investigate the age-related expression change on BMSCs, 2- 8- and 22-month BMSCs proteins were extracted. Consistent with the trend in the cranial bone, a reduction in BMAL1 expression with aging was observed on BMSCs (Fig. 1f, g). These findings suggest that a decrease in BMAL1 expression may significantly affect bone aging and homeostasis. Next, the temporal expression of the circadian clock gene Bmal1 in 2-month-old and 22-month-old BMSCs was assessed by qRT-PCR and western blot analysis (Fig. 1h). Significantly postponed and blunted circadian rhythms were observed in 22-month-old BMSCs compared to 2-month-old BMSCs at protein level (Fig. 1h, i). It is worth noting that the postponement of fluctuation was evident at the protein level, whereas at the transcriptional level, there was mainly a decrease in overall Bmal1 expression. These results demonstrated that posttranscriptional regulation may be involved in coordinating of age-related circadian rhythms in BMSCs. To explore the age-related changes of molecular rhythms in BMSCs during the osteogenic process, after seven days of osteogenic induction, the cells were synchronized by 2 hr pulse treatment and harvested at 6 h intervals. The mRNA levels of *Bmal1, Clock, Per2*, and *Rev-erbα* revealed drastic changes in aged BMSCs compared to young cells (Fig. 1j). The normal circadian rhythm was disrupted with aging.

### Age-dependent miR-142-3p increased with aging and altered osteogenic capability in BMSCs by targeting Bmal1

**Fig. 2:**
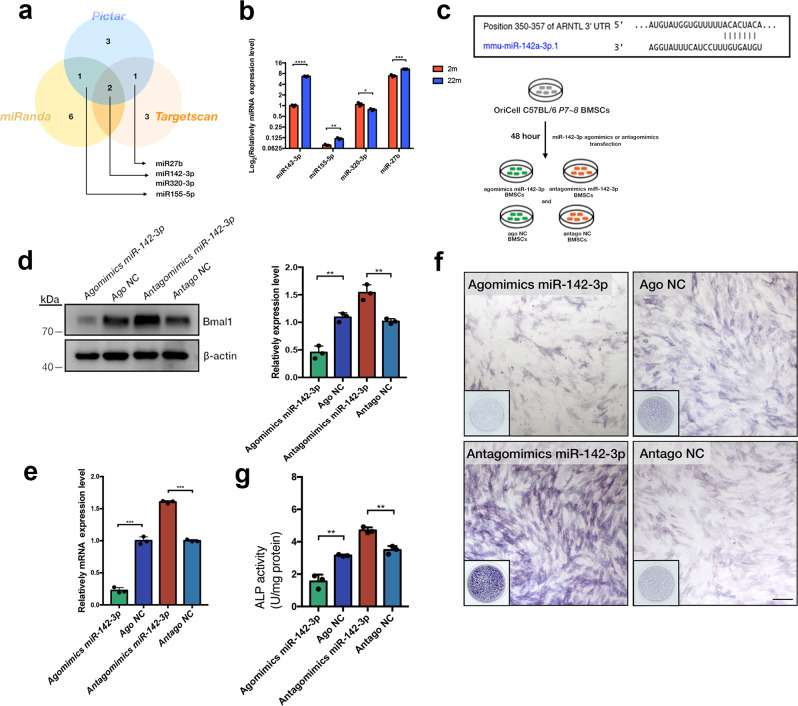
Age-dependent miR-142-3p downregulates the clock gene Bmal1 and alters osteogenic capability in BMSCs. **a** Venn diagram showing predicted miRNAs targeting Bmal1 with three bioinformatic algorithms (TargetScan, Pictar and miRanda). **b** qRT-PCR analysis of the expression of predicted miRNAs (miR142-3p, miR155-5p, miR320-3p and miR-27b) in 2-month-old and 22-month-old BMSCs. **c** Predicted miR-142-3p binding site on Bmal1 3’UTR and workflow diagram of miR-142-3p agomimics and antagomimics transfection in OriCell C57/BL6 BMSCs. The cells were named Agomimics miR-142-3p, Ago NC, Antagomimics miR-142-3p and Antago NC. **d** Western blot and quantitative analysis of Bmal1 expression 48 h after transfection of miR-142-3p agomimics and antagomimics. **e** qRT-PCR analysis of mRNA expression of *Bmal1* after 48 h transfection of miR-142-3p agomimics and antagomimics. **f** ALP staining at day 7 of osteogenic induction after transfection of miR-142-3p agomimics and antagomimics. Scale bar: 200 μm. **g** Quantitative analysis of data f. Transfected group was compared with mock group. *N* = 3, Data are presented as mean ± SD; *p < 0.05, **p < 0.01, ***p < 0.001 by two-tailed Student’s *t*-tests.

To investigate the possible posttranscriptional regulation mechanism, circadian miRNAs that targeted the clock gene Bmal1 were screened. Computational prediction was performed by three algorithmic methods, and four high-scored miRNAs were screened out (miR-142-3p, miR-155-5p, miR-320-3p, and miR-27b) that putatively target Bmal1 (Fig. 2a). The expression of miR-142-3p was the most drastically increased among the four miRNAs in 22-month BMSCs compared to 2-month BMSCs (Fig. 2b). Therefore, miR-142-3p was identified as the age-dependent circadian miRNA targeting the clock gene Bmal1 in BMSCs. To assess the functional alteration of miR-142-3p on cell proliferation and osteogenic capability, agomimics miR-142-3p, antagomimics miR-142-3p and mock vectors were constructed according to the predicted targeting site on Bmal1 3’UTR sequence (Fig. 2c). The OriCell C57BL/6 BMSCs were used in this experiment. The RNA transfection workflow diagram was shown in Fig. 2c. It was shown that transfection of a miR-142-3p agomimic reduced Bmal1 expression in mRNA and protein level, whereas transfection with a miR-142-3p antagonist elevated Bmal1 expression (Fig. 2d, e). ALP staining, which was performed after 7-day osteogenic induction, suggested that the downregulation of miR-142-3p promoted BMSC osteogenic differentiation, and the upregulation of miR-142-3p inhibited BMSC osteogenic capability (Fig. 2f, g). However, the CCK-8 assay at 2-day intervals after RNA transfection showed that loss or gain of miR-142-3p in BMSCs did not affect cell proliferation (Fig. S1).

### miR-142-3p/inhibitor BMSCs exerted similar change on molecular rhythm and osteogenic capacity with Bmal1/OE BMSCs

**Fig. 3:**
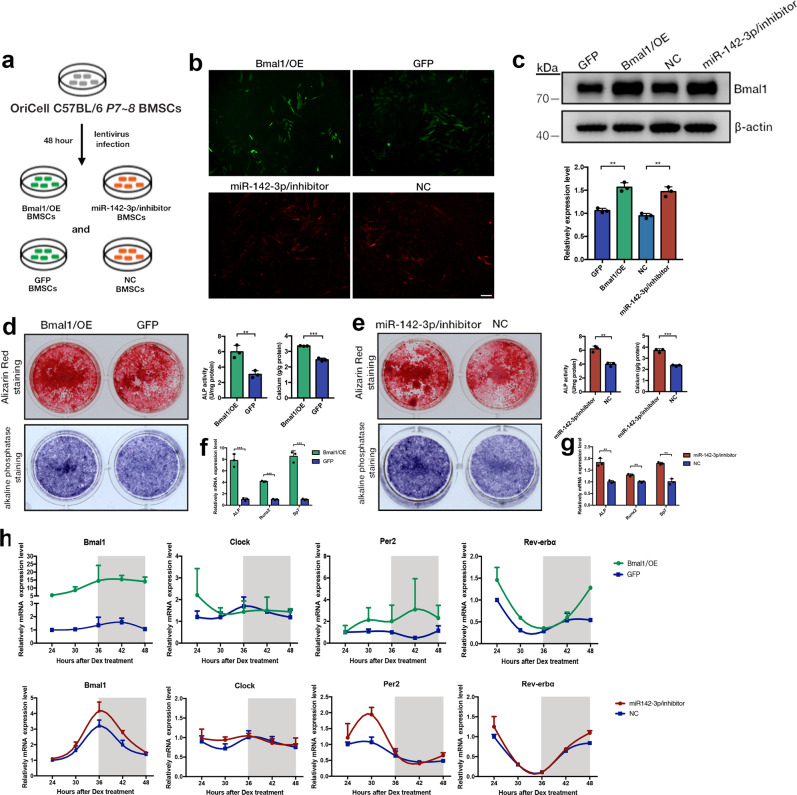
Transcriptional and posttranscriptional upregulation of Bmal1 by Bmal1/OE and miR-142-3p/inhibitor promotes osteogenic capability and rescues circadian oscillation in BMSCs. **a** Workflow diagram of lentivirus transfection in OriCell C57/BL6 BMSCs. The cells were named Bmal1/OE, GFP, miR-142-3p/inhibitor and NC. **b** Fluorescence microscopy of BMSCs after lentivirus transfection for 48 hours. Scale bar: 50 μm. **c** Western blot and quantitative analysis of Bmal1 expression in Bmal1/OE, GFP, miR-142-3p/inhibitor and NC BMSCs after transfection for 48 h. Overexpressed/inhibited group was compared with the mock group. **d**, **e** Representative pictures and quantitative analysis of alkaline phosphatase staining (ALP) and Alizarin red staining (ARS) at 7 d and 14 d on Bmal1/OE, GFP and miR-142-3p/inhibitor, NC BMSCs respectively. **f**, **g** qRT-PCR analysis of the osteogenic-related gene expression (*Alp, Run2, Sp7*) under osteogenic conditions in Bmal1/OE, GFP and miR-142-3p/inhibitor, NC BMSCs respectively. **h** qRT-PCR analysis of molecular rhythmic expression of circadian clock genes (*Bmal1, Clock, Per2, Rev-erbα*) in Bmal1/OE, GFP, miR-142-3p/inhibitor and NC BMSCs under osteogenic conditions. *N* = 3, Data are presented as mean ± SD; **p < 0.01, ***p < 0.001 by two-tailed Student’s *t*-tests.

To elucidate the potential functional differences of Bmal1 upregulation at the transcriptional and posttranscriptional levels, lentiviral vectors were transfected in BMSCs with Bmal1 over-expression and miR-142-3p inhibition, labeled with eGFP and mCherry, respectively (Fig. 3a). Vector information of Bmal1 over-expression and miR-142-3p inhibition plasmids were shown in Fig. S2a. Transduction efficiencies were verified by fluorescence microscopy and western blots (Fig. 3b, c). We next investigated the osteogenic capability of Bmal1/OE and miR-142-3p/inhibitor BMSCs, as evidenced by alkaline phosphatase staining (ALP) and alizarin red staining (ARS) (Fig. 3d, e). Significant increases in ALP activity and calcium mineralization were observed in Bmal1-upregulated and miR-142-3p-inhibited BMSCs. Coincident with the ALP and ARS staining results, the mRNA levels of osteogenic markers in Bmal1/OE and miR-142-3p/inhibitor BMSCs increased under osteogenic conditions, as confirmed by qRT-PCR (Fig. 3f, g). Senescence-positive cells were significantly decreased after Bmal1 over-expressed, along with a decrease in p16^ink4a^ and p21^waf1/cip^ expression. But in miR-142-3p/inhibitor BMSCs, the count of positive senescence cells and p16^ink4a^ expression was not significantly altered, while p21 ^waf1/cip^ was upregulated (Fig. S2b, c). We further investigated the molecular rhythm alterations in Bmal1/OE and miR-142-3p/inhibitor BMSCs under osteogenic conditions. qRT-PCR analysis revealed a recovery of molecular rhythms for the core circadian gene Bmal1 and Clock and classic BMAL1 target clock-controlled genes (*Rev-erbα* and *Per2*) after Bmal1/OE and miR-142-3p/inhibitor transduction (Fig. 3h). In summary, transcriptional or posttranscriptional upregulation of the clock gene Bmal1 could promote osteogenic capability and rescue molecular rhythm in BMSCs.

### Bmal1 promotes BMSCs osteogenesis by deregulating YAP, which is independent of the activation of Hippo pathway

**Fig. 4:**
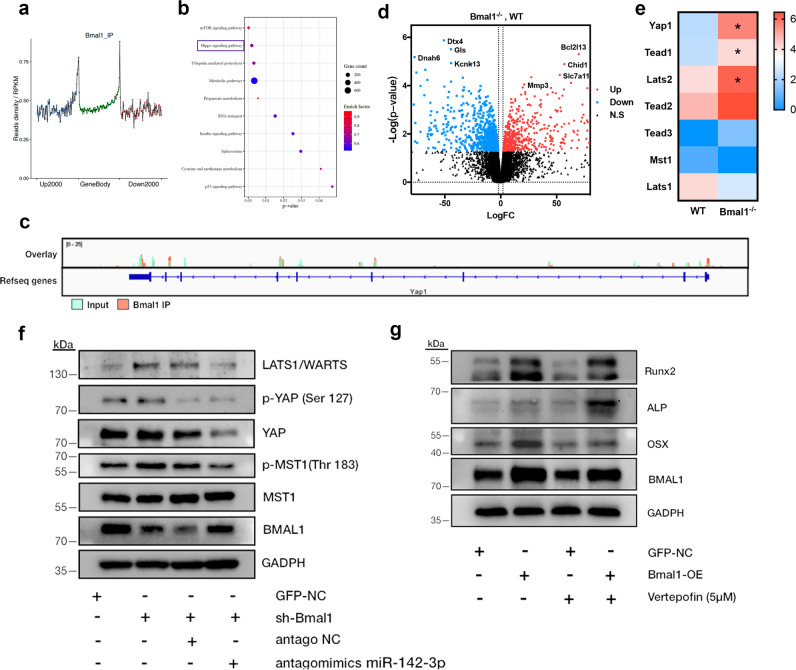
Bmal1 promotes BMSCs osteogenesis via miR142-3p/Bmal1/YAP axis. **a**, **b** Bmal1 binding regions related to the gene body and enriched KEGG pathways for Bmal1 mediated biological process by ChIP-seq. The box indicated the enriched Hippo pathway (*p* = 0.002, enrich factor = 0.668). **c** ChIP tracks viewed by the integrated Genome Viewer (IGV) revealed that Bmal1 has high enriched peak at YAP coding region. **d** Volcano map of differential expressed mRNAs profile in Bmal1−/− and wildtype BMSCs, based on GSE106586 dataset. **e** Heat map of Hippo pathway genes expression in Bmal1−/− and wildtype BMSCs. *p < 0.05. **f** Western blot analysis of Hippo pathway components (LATS1/WARTS, p-MST1, MST1, p-YAP, YAP) in Lv-shRNA-Bmal1 and mock vectors (short for sh-Bmal1 and GFP-NC) transfected OriCell C57/BL6 BMSCs, then rescued by transfected miR142-3p antagomimics. **g** Western blot analysis of osteogenesis related genes (RUNX2, ALP, OSX) in Bmal1/OE and mock vectors transfected OriCell C57/BL6 BMSCs and Verteporfin (5 μM) treated cells after five days osteogenic induction.

It is well known that Bmal1 coordinates circadian oscillation by transcriptional activation of clock-output genes [14, 15, 21]. To further understand the molecular mechanism underlying the regulation of Bmal1 on BMSCs osteogenesis, we performed ChIP-sequence to detect the potential binding region of Bmal1 on BMSCs. Figure 4a showed that read density was high at 1000 bp upstream and downstream of transcriptional start site (TSS) and transcriptional terminal site (TTS) region, where TFs bind and act as regulated roles in the transcription process. Enriched KEGG pathways of the Bmal1 regulated biological process showed that metabolic pathways, mTOR pathway and Hippo pathway were the most enriched pathways (Fig. 4b). Focus on the Hippo pathway (*p* = 0.002, enrich factor = 0.668), the enriched peak of BMAL1 was found on the TTS region of Yap1 using IGV tools (Fig. 4c). We further mined the GSE106586 RNA-seq dataset to screen the mRNAs expression profile on Bmal1-knockout and wildtype BMSCs. A total of 849 genes were defined as differentially expressed and shown in the Volcano map (Fig. 4d). Fold changes of Hippo pathway core genes were confirmed and showed with heatmap (Fig. 4e). Collectively, the Hippo pathway was considered as the potential downstream pathway involved in Bmal1 regulation of BMSCs osteogenic lineage commitment. To investigate whether Hippo signaling pathway is regulated by the miR-142-3p/Bmal1 axis and affected BMSCs osteogenesis, rescue experiments were performed to confirm the biological role of miR-142-3p/Bmal1/Hippo signaling. In our previous study, lenti-GFP and lenti-shRNA-Bmal1 lentiviral vectors (short for GFP-NC and sh-Bmal1 respectively) had been constructed and verified to decrease the expression of Bmal1 in BMSCs [22]. Here, we inhibited Bmal1 by transfection of sh-Bmal1 and rescued it with miR-142-3p antagomimics transfection. As shown in Fig. 4f, the expression of LATS1/WARTS was increased and phosphorylated level of the core molecule MST1 was elevated. The silence of miR-142-3p by miRNA transfection rescues the alteration of LATS1/WARTS, p-MST and YAP by inhibiting Bmal1 expression. However, the expression of phosphorylated YAP was upregulated after Bmal1 inhibition. Additionally, miR-142-3p inhibition didn’t decrease the phosphor-YAP (Ser 127) level, which should be degraded when Hippo pathway was inactive. Therefore, other regulation mechanisms may involve this miR-142-3p/Bmal1 mediated process and feedback system may be existed on Bmal1 regulation of YAP through Hippo pathway. To further verify the effect of Bmal1 mediated YAP expression on BMSCs osteogenesis, Vertepofin, a YAP/TEAD binding inhibitor were used to downregulate YAP expression. As shown in Fig. 4g, Bmal1/OE BMSCs exhibited promoted osteogenic capability compared to GFP-NC cells. Treated with Vertepofin, the expression of all osteogenic markers in BMSCs was reduced. It was possibly due to cytotoxicity and multiple functional roles of Veterpofin on cultured cells [23]. Compared with Vertepofin treated GFP-NC BMSCs, the Bmal1-OE BMSCs presented greater osteogenic capability in Vertepofin supplemented medium, which verifies the effect of Bmal1 on BMSCs osteogenesis. These results suggested that Bmal1 promoted BMSCs osteogenesis by deregulation of YAP, but this process was independent from inactivated of Hippo pathway. Conversely, Bmal1 elevated the phosphor-level of Hippo pathway core components MST1 and expression of LATS1/WARTS. This contradicted result could be explained by the Bmal1 directly transcriptional regulation on YAP1, and miR-142-3p may further participate in the process of YAP phosphorylation.

### Transplantation of Bmal1/OE and miR-142-3p/inhibitor BMSCs into cranial bone defects promoted bone formation and turnover in aged-mice

**Fig. 5:**
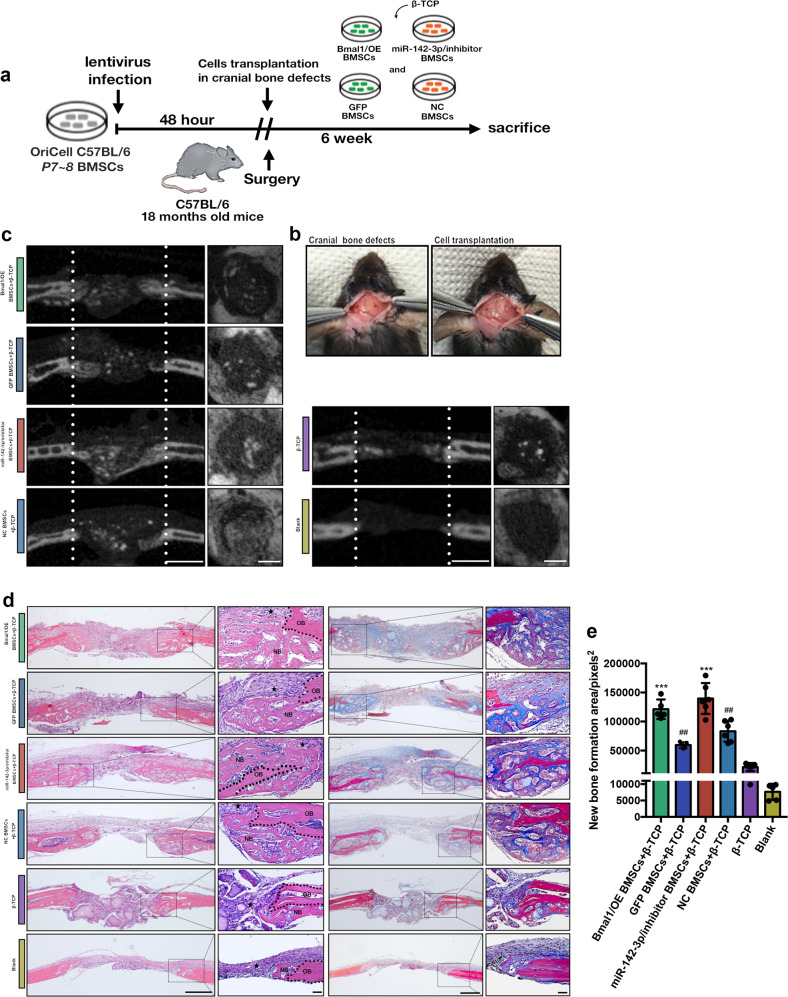
Bmal1/OE and miR-142-3p/inhibitor BMSCs promotes bone formation in cranial defects of aged mice. **a** Flow chart diagram of the cell transplantation process. **b** Photograph of animal surgery and cell transplantation procedure in mice. **c** micro-CT images of the cells transplanted defect sites. Scale bar: 500 μm. **d** H&E staining (H&E) and Masson trichrome staining (Masson) of cells transplanted defect sites. NB represents new bone, OB represents old bone, and represents connective tissue. Scale bar: 100 μm for 4x images, 50 μm for 10x images. **e** Quantitative analysis of new bone formation area according to Masson staining. *N* = 5–6; Data are presented as mean ± SD; ***p < 0.001 compared with the mock group, ##*p* < 0.01 compared with the blank group by one-way ANOVA with Tukey’s post hoc test.

**Fig. 6:**
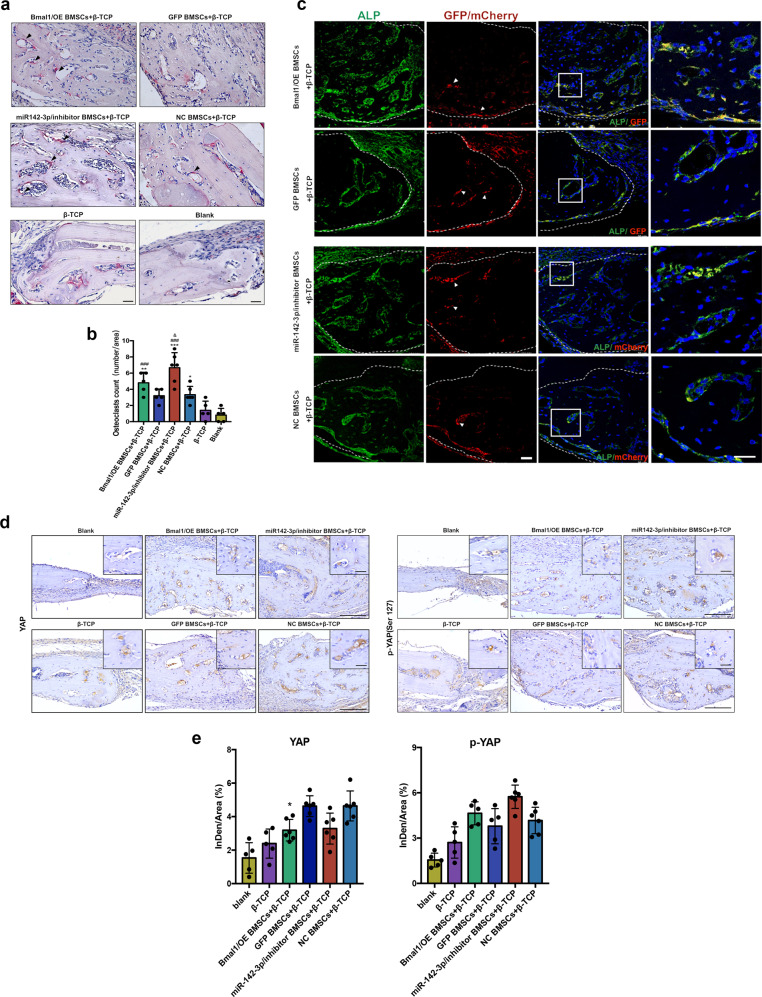
High bone turnover activity and phospho-YAP level are present in Bmal1/OE and miR-142-3p/inhibitor BMSC-transplanted cranial defects in aged mice. **a** TRAP staining and quantitative analysis of the number of osteoclasts at transplanted defect sites; arrows indicate TRAP+ cells. Scale bar: 25 μm. **b** Quantitative analysis of in data a. **c** Double immunostaining images of ALP-expressing and GFP+ or mCherry+ cells, with nuclei stained with DAPI; arrows indicate GFP/mCherry+ cells. Scale bar: 30 μm. **d** Immunostaining images of p-YAP, YAP in cells transplanted defect sits. Scale bar: 100 μm for 4x images, 25 μm for 40x images. **e** Quantitative analysis of data in d. *N* = 5–6; Data are presented as mean ± SD; (**b**) *p < 0.05, **p < 0.01, ***p < 0.001 compared with the blank group, ###*p* < 0.001 compared with β-TCP group, & *p* < 0.05 compared with the mock group by one-way ANOVA with Tukey’s post hoc test. (**e**) *p < 0.05 compared with mock group by one-way ANOVA with Tukey’s post hoc test.

To confirm that miR-142-3p and Bmal1 promote osteogenic capability in aged BMSCs in vivo, clock-modified BMSCs were transplanted into cranial bone defects in aged mice (Fig. 5a, b). Micro-CT images showed that the blank and β-TCP groups presented barely any bone formation (Fig. 5c). New bone formation was evident in the Bmal1/OE and miR-142-3p/inhibitor groups compared with the GFP and miRNA/NC groups (Fig. 5d). Histological analysis showed that larger new bone area was observed in clock-modified BMSCs groups, which confirmed the osteogenic capability of Bmal1-overexpressing and miR-142-3p-inhibited BMSCs in vivo (Fig. 5e). TRAP staining showed that osteoclasts at the border of the new bone were highly active in the Baml1/OE and miR-142-3p/inhibitor groups (Fig. 6a, b). Immunostaining demonstrated that more ALP-expressing cells appeared in the new bone in the Bmal1/OE and miR-142-3p groups than in the other groups, and GFP+ cells and mCherry+ cells indicate Bmal1/OE and miR-142-3p/inhibitor BMSCs, respectively (Fig. 6c). In addition, the transplanted cells showed strong ALP expression near the newly formed bone, which indicated that the donor cells might also contribute to osteoblast lineage commitment in vivo. We further performed IHC analysis to investigate the expression level of YAP and p-YAP in newly generated bone. As shown in Fig. 6d, e, the expression level of YAP were significantly decreased in miR-142-3p/inhibitor and Bmal1/OE group at the new bone area, which further confirm the results of ex vivo experiments. In summary, the results showed that transplanted clock-modified BMSCs improved bone formation, and accelerated bone repair by inhibiting of YAP expression in aged mice.

### Intra-femoral injection of Bmal1/OE and miR-142-3p/inhibitor BMSCs ameliorates osteoporosis in aged mice

**Fig. 7:**
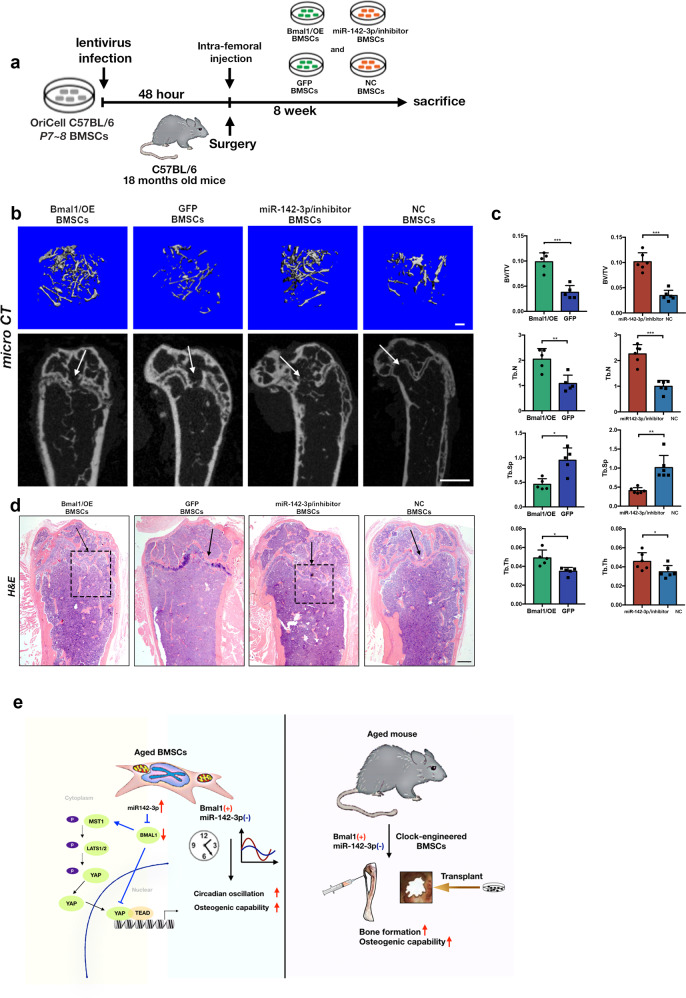
Supplementation of Bmal1/OE and miR-142-3p/inhibitor BMSCs intra-femur enhance bone deposition in aged mice. **a** Flow chart diagram of intra-femoral clock-modified BMSCs delivery. **b** Representative 3D constructed and sectional images of the metaphyseal region of recipient femurs. Scale bar: 200 μm for 3D images and 1 mm for sectional images. The arrows indicated the direction of cells delivery. **c** Quantitative micro-CT analysis (BV/TV, Tb.N, Tb.Th, Tb.Sp) of the injected region of recipient femurs. **d** H&E staining shows bone volume on recipient femurs. Scale bar: 200 μm. The arrows and boxes indicated the injection site and assumed regenerating area. **e** Graphical summary of mechanisms underlying the clock-modified BMSCs therapy on aged mice via the miR-142-3p/Bmal1/YAP signaling axis. *N* = 5–6, Data are presented as mean ± SD; *p < 0.05, **p < 0.01, ***p < 0.001 by two-tailed Student’s *t*-tests.

To further test the efficacy of modified cell therapy for aged mice, an intra-femoral transplantation model was used to determine whether the infusion of Bmal1/OE and miR-142-3p/inhibitor into femurs enhanced bone deposition in aged mice as previously described [24] (Fig. 7a). The micro-CT data revealed that the Bmal1/OE and miR-142-3p/inhibitor groups exhibited greater regeneration at the metaphyseal femur sites than the control groups (Fig. 7b). The femurs injected with clock-modified BMSCs showed more mineralization deposition than those with control cells injected into the cavities. Quantitative micro-CT analysis confirmed the increased bone volume and amount of trabecular bone in the cell hosted region in the Bmal1/OE and miR-142-3p/inhibitor groups compared with the control groups (Fig. 7c). Figure 7d showed the hematoxylin and eosin (H and E)-staining analysis of recipient femurs. The box areas indicate putative cells hosting the metaphyseal region. Representative images show that the Bmal1/OE and miR-142-3p/inhibitor groups presented relatively extensive trabecular bone compared with the control groups. The above results further confirmed that the delivery of clock-modified BMSCs contributes to bone formation in recipient femurs in aged mice. Above all, the Fig. 7e show the graphical summary of the mechanism underlying the clock-modified BMSCs therapy on aged mice via the miR-142-3p/Bmal1/YAP signaling axis.

## Discussion

With an increasingly aging population, age-associated diseases and potential effective therapeutic treatments have attracted particular attention. It is well recognized that the circadian rhythm deteriorates with age and leads to a series of changes in biological behavior and tissue physiology [25, 26]. A low-bone-mass phenotype was identified in Bmal1 knockout mice and Bmal1 osteoblast lineage deletion mice, highlighting the clock system’s essential importance in better understanding bone biology [12, 27]. However, previous studies have less investigated possible translational applications related to the circadian rhythm’s effects on bone regeneration. Two strategies could be applied to facilitate tissue engineering and homeostasis. One is the delivery of drugs directly targeting the clock genes and CCGs in vivo, which may promote regeneration after damage. Another could be molecular engineering of core clock genes in cultured stromal cells ex vivo to synchronize the circadian clock and engraft on the defects. Here, the current study is the first to report the usage of clock-enhancing BMSCs in the treatment of age-related bone loss. Given the reduced BMAL1 levels and dampened daily cellular rhythms observed in aged bone and BMSCs, we confirmed the essential role of core clock factors in the balance of bone homeostasis with aging. Therefore, it is reasonable to predict that the core circadian gene Bmal1-induced peripheral oscillation may be a significant contributing factor for the maintenance of bone mass in tissue engineering medicine.

In addition to canonical transcriptional feedback regulation, the posttranscriptional modification also plays a critical role in the circadian oscillation of the peripheral clock [28–30]. Several miRNAs were reported to involve in circadian rhythm and shape the tissue-specific circadian proteome [21, 31]. miR-219 and miR-132 are brain-specific miRNAs that participate in rhythm length regulation and light-induced circadian resetting [16, 17]. Unlike the heterodimer directly binding to the E-box in the upstream regulatory sequence, miRNAs competitively bind to the mRNA 3’UTR of targeted clock genes, which could have different effects on downstream biological reactions. Nevertheless, limited evidence has been reported about the bone- or BMSC-specific miRNAs that regulate circadian rhythms, how they affect bone homeostasis and how they affect the different regulatory roles of circadian rhythms at the transcriptional and posttranscriptional levels. Interestingly, this study, demonstrated the obvious shift of circadian rhythm between mRNA and protein expression level with aging, including postpone in amplitudes and phases. This founding suggested the critical role of posttranscriptional regulation in circadian timing and stem cells aging. We also identified miR-142-3p as an age-dependent miRNA that targets the core clock gene Bmal1 and contributes to the robustness of the circadian rhythm in BMSCs. Of note is that we observed that some alterations in phenotype in the miR-142-3p/inhibitor group were different from those seen in the Bmal1/OE group in terms of the effects on SASP expression and the oscillation of CCGs. This suggests that other mechanisms may be involved in this circadian regulation network. One explanation could be that miRNAs are multiply targeted to the 3’UTR sequence. Other genes, including Foxo1 and Nr3c1, have also been predicted to be regulated by miR-142-3p [32, 33].

Recent studies have suggested that the circadian clock may be implicated in regenerative medicine and play a crucial role in the wound healing response and bone regeneration in mice and humans [34–36]. Holye et al. reported that clock-controlled cytoskeletal proteins involved in cell migration participate in the wound-healing response [34]. Mengatto et al. performed genome-wide transcriptomic analysis of the jawbone implant failure model, which showed that circadian rhythm is one of the top molecular pathways affected [37]. BMSCs display their own peripheral rhythm, influencing multiple factors and affects bone regeneration. Hassan reported that titanium-based biomaterials could suppress the expression of *Per1* on BMSCs, consequently altering the circadian rhythm of BMSCs and diminishing the rate of osseointegration [38]. These studies highlight that peripheral circadian signaling could be a potential novel target for tissue regeneration and translational medicine. Our study, demonstrated that the temporal expression of clock output genes could be reversed in BMSCs by upregulating the core circadian gene Bmal1 at both the transcriptional and posttranscriptional levels.

Hippo pathway is known as the potent signaling pathway regulating numerous biological processes. Recent research demonstrated that the Hippo pathway acts as a crucial positive regulator on maintaining intestinal stem cell (ISC) niche and increasing ISC clock activities [39]. The function of the Hippo pathway in clock-mediated bone homeostasis has not been investigated. As the terminal transcriptional activator, studies on YAP/TAZ in bone homeostasis have shown conflicting evidence, indicating the regulatory mechanism in mesenchymal progenitor cell differentiation is far more complicated in vivo and *vitro* [40]. Seo observed that SOX2 targeted YAP and inactivated WNT/β-catenin signaling to inhibit osteogenic differentiation in BMSCs [41]. On the contrary, Pan reported OB-lineage YAP knocking out Ocn-cre; YAP mice showed trabecular bone loss but normal cortical bone volume [42]. The latest study showed that YAP1/TAZ constrains the expression of hypoxia-sensitive genes and negatively regulates angiogenesis and osteogenesis [43]. These results suggested that Hippo/Yap signaling pathway may act differently in the organ-dependent and temporal-associated pattern. Here, by analyzing ChIP-seq and RNA-seq datasets, we reported that transcription factor YAP was under the regulation of core circadian gene Bmal1 and negatively regulated osteogenesis. However, as the upstream negative regulation signaling on YAP, Hippo pathway core components were activated in Bmal1-inhibited BMSCs, which suggested that multiple mechanisms may account for this process. One explanation was that Bmal1 could directly transcriptionally regulate YAP supported by ChIP-seq, which may consist of a feedback loop system on Bmal1-regulated YAP expression. Additionally, ex vivo and in vivo experiments verified that inhibition of miR-142-3p decreased the phosphor-level and expression of upstream Hippo pathway core genes, but elevated the expression of p-YAP (Ser 127), which suggests the regulatory role in the phosphorylation and nucleus transportation of YAP.

Our clock-enhanced therapy highlighted the crucial role of circadian rhythm in regeneration medicine and demonstrated a potential post-translational target on circadian therapy. However, our study unrevealed the dynamic of bone metabolism and related biological processes during circadian disruption. Therefore, future investigations will be needed before taking these circadian-related therapies to the clinic.

In summary, our study demonstrates that overexpression of Bmal1 or inhibition of miR-142-3p rescues the abolished peripheral circadian rhythm and osteogenic capability in aged BMSCs and promotes bone formation in aged mice in vivo by inhibiting YAP expression. This pilot application provides new therapeutic avenues for circadian rhythm-guided bone tissue engineering.

## Conclusion

This study demonstrated that age-dependent miR-142-3p could downregulate Bmal1 at the posttranscriptional level and weaken the osteogenic differentiation of BMSCs through up-regulation of YAP, which was independent from the inactivation of Hippo pathway. The decreased osteogenic capability of BMSCs can be reversed by an increased expression of core circadian clock gene Bmal1 and the robust peripheral circadian rhythm. Transplantation of clock-engineered BMSCs in aged mice promotes bone regeneration and reverses age-related bone loss.

## Materials and methods

### Animal

Male C57BL/6 mice at 2-month, 8-month and 18–22-month of age were purchased from the Experimental Animal Center of Sichuan University and housed in cages under standard animal housing conditions with a 12-hour light and 12-hour night cycle. All mouse experiments were approved by the animal ethics committee of Sichuan University (WCHSIRB-D-2018-168).

### Cell culture

Primary 2-month, 8-month and 22-month BMSCs were isolated by flushing the bone marrow of the femurs and tibiae as previously described [44]. The cells were then cultured in α-MEM medium (Gibco) supplemented with 10% fetal bovine serum (Gibco) and 1% penicillin and streptomycin (HyClone) in a 5% CO_2_ atmosphere at 37 °C. Primary BMSCs were used for the experiments at passages 2. OriCell C57/BL6 BMSCs at passage 5 were purchased from Cyagen (Cyagen, Guangdong, China) and cultured in OriCell growth medium (Cyagen) as manufacturer’s instructions. For miRNA agomimics and antagomimics transfection, lentivirus transfection and BMSCs transplantation, OriCell C57/BL6 BMSCs at passage 7–8 was used in these experiments.

For BMSCs osteogenic differentiation, cells were induced by osteogenic conditional medium when reached 70% confluence. The osteogenic culture medium containing α-MEM medium (Gibco) supplemented with 50 μM ascorbic acid, 10 mM β-glycerophosphate and 10 nM dexamethasone (Sigma-Aldrich). Culture medium was changed every two days. For BMSCs adiopogenic differentiation, cells were induced by adipogenic conditional medium when reached 90% confluence. The adipogenic medium containing DMED medium supplied with 1 mM dexamethasone, 10 nM insulin and 200 mM indomethacin (Sigma-Aldrich).

### RNAs and lentivirus transfection

For miRNAs transfection, the miR-142-3p agomimics, miR-142-3p antagomimics and mock vectors were constructed by Hanbio Co., Ltd, Shanghai, China. Prior to transiently transfection, the OriCell C57/BL6 BMSCs at passage 7–8 were cultured in 12-well plates. Transiently transfected were using Lipofectamine 3000 reagent (Invitrogen) according to the manufacturer’s protocol. The transfected cells were named Agomimics miR-142-3p, Ago NC, Antagomimics miR-142-3p and Antago NC respectively.

For lentivirus transfection, the lentiviral vectors for Lv-GFP-Bmal1, Lv-mCherry-inhibitor-miR142-3p, and mock vectors were purchased from GenePharma Co., Ltd, Shanghai, China. The vectors information was shown in Fig. S2a. Prior to cells transfection, the OriCell C57/BL6 BMSCs at passage 7–8 were seeded in 6-well and 12-well plates. When the cells confluence reached 40-50%, cells were transfected with lentiviral particles and experiments were performed 48 h after transfection. The transfected cells were named Bmal1/OE, GFP, miR-142-3p/inhibitor and NC respectively.

For the gain- and loss- function experiment, the lentiviral vector Lv-shRNA-Bmal1 and mock vectors (short for sh-Bmal1 and GFP-NC) were used to decrease Bmal1 expression, which has been illustrated and verified in our previous study [22]. The OriCell C57/BL6 BMSCs at passage 7–8 were used in this experiment and the transfection process was as previous description. To rescue the effect of Bmal1 downregulation, miR-142p-3p antagomimics and mock vector were transfected 48-hour following Lv-shRNA-Bmal1 transfection and cells were harvested 72 h after antagomimics transfection for western blot analysis.

To explore the functional role of Bmal1/YAP in osteogenesis, the lentiviral vector Lv-GFP-Bmal1 and mock vectors were transfected in OriCell C57/BL6 BMSCs at passage 7-8, and Verteporfin (5 μM, #HY-B1046, MedChemExpress) was added to inhibited YAP expression 48 hours after lentivirus transfection. Then the cells were induced in osteogenic medium for 5 days and harvested for western blot analysis.

### Circadian rhythmicity of clock genes expression determination

To explore the rhythmic expression of circadian clock genes in 2-month and 22-month BMSCs, primary BMSCs in two age groups were isolated from mice according to Zeitgeber Time at 6-hour intervals (ZT0, ZT6, ZT12, ZT18, ZT24). The cells were harvested for RT-qPCR and western blot analysis at passage 2.

To assess the molecular rhythms of the circadian genes under osteogenic condition, the BMSCs were induced in osteogenic medium for 5 days, then the cells were synchronized and harvested as described previously [45]. Briefly, the cells were synchronized by a 2-hour pulse treatment with 0.1 μM dexamethasone, then the recording complete medium was changed, and RNA of cells were extracted for RT-qPCR analysis at 6-hour intervals after 24 hours.

### RNA extraction and RT-qPCR

RNA was extracted from the cells with TRIzol (Invitrogen) and isolated with a standard isopropanol and ethanol procedure. The RNA concentration was measured with the NanoDrop 2000 (Thermo Fisher Scientific) and then reverse transcribed into cDNA using a PrimeScript RT reagent Kit with gDNA Eraser (Takara) for mRNA and the Mir-X miRNA First Strand Synthesis Kit (Takara) for miRNA. RT-qPCR was performed using SYBR Premix Ex Taq II (Takara) on an Applied Biosystems Quant Studio 7 (Thermo Fisher Scientific). Relative gene expression was normalized by *Gadph* for mRNA expression and *U6* for miRNA expression using the 2^−ΔΔCt^ method. The primers are listed in Additional file Table S1.

### Alkaline phosphatase activity assay and Alizarin red staining

The cells were induced with osteogenic differentiation medium for 7 days before ALP staining and 14 days before ARS staining. Then cells were fixed with 4% polyoxymethylene for 15 min. For ALP staining, the fixed cells were incubated with 0.1 M Tris buffer containing 0.25% naphthol AS-BI phosphate and 0.75% Fast Blue for 20 min. The quantitative ALP activity was analyzed by the alkaline phosphatase assay kit (Beyotime) and optical density was measured by the spectrophotometer (Thermo Fisher Scientific) at 405 nm.

For ARS staining, the fixed cells were stained with 1% Alizarin red S (pH 4.2, Sigma-Aldrich) for 10 min. The mineralized was destained by 10% cetylpyridinum in 10 mM sodium phosphate (pH 7.0). Calcium concentration was detected by the spectrophotometer (Thermo Fisher Scientific) at 562 nm with standard calcium curve in this solution.

### Western blot

Tissues and cells were lysed in ice-cold RIPA buffer (Sabbiotech). Proteins were separated by SDS-PAGE, transferred to PVDF membranes and blocked with 5% milk for 1 h. Membranes were incubated with rabbit anti-BMAL1 (1:1000, #14020, CST), anti-beta actin (1:1000, #8457 S, CST), anti-Runx2 (1:1000, #ab236639, Abcam), anti-ALP (1:500, #ab229126, Abcam), anti-GADPH (1:1000, #ab8245, Abcam), anti-OSX (1:1000, #ab209484, Abcam), anti-YAP (1:1000, #1407 S, CST), anti-p-YAP (1:1000, #13008, CST), anti-MST1 (1:1000, #3682 S, CST), anti-p-MST1 (1:1000, #49332 S, CST), anti-LATS1/WATRS (1:1000, #ab243656, Abcam) 4 °C overnight, followed by a horseradish peroxidase-conjugated secondary antibody (1:5000, #L3012-2, Sabbiotech). Signals were visualized using an ECL detection kit (Thermo Fisher Scientific).

### Colony-forming units-fibroblasts (CFU-F) and cell proliferation assay

Primary cells were seeded in 6-well plates at 1000 cells/per well. The medium was changed at 3-day intervals, and the formation of colonies was assessed after 14 days of culture. The cells were fixed with 4% paraformaldehyde and stained with crystal violet. Colonies with >50 cells were counted under light microscopy.

Cell proliferation of transfected cells was assessed by a Cell Counting K-8 assay (CCK-8) kit according to the manufacturer’s instructions (Beyotime Biotechnology, Shanghai, China). Briefly, after miR-142-3p agomimics, antagomimics and mock vectors transfection, the transfected cells were seeded on 96-well plates in a 1000 cells per well density. CCK-8 reagent was added into the culture medium and incubated in 37 °C for 2 hours. Optical absorbance was measured by the spectrophotometer (Thermo Fisher Scientific) at 450 nm when cells were cultured 1-day, 3-day, 5-day and 7-day.

### Assessment of senescence-associated β-galactosidase staining (SA β-gal)

Positively senescent cells were assessed by a senescence-associated β-galactosidase staining kit (Cell Signaling Technology) according to the manufacturer’s protocol. Briefly, the cultured cells were fixed with paraformaldehyde and then incubated at 37 °C overnight with β-gal staining solution. Positive blue-stain cells were counted under microscopy.

### Bioinformatics and data analysis

For Bmal1 targeting miRNAs screening, the bioinformatics algorithms miRanda (Computational Biology Center, NY, USA), TargetScan (David Bartel Lab, MA, USA) and PicTar (Rajewsky lab, NY, USA and Max Delbruck Centrum, Berlin, DE) were applied.

The GSE106586 dataset was used to analysis the RNA expression profiles of Bmal1-knockdown and wildtype (WT) BMSCs. The limma package of *R* was used to identified the differential RNA expression on BMSCs of Bmal1-/- and WT. Genes were considered as significant if *p* value lower than 0.05 and |logFC | å 1 and showed with volcano map.

### ChIP-sequencing

The ChIP assay for the OriCell C57/BL6 passage7 BMSCs cells were performed with BMAL1 (1:10, #14020, CST) using a SimpleChIP Assay Kit (#9003 Cell Signaling Technology). The input and Bmal1 immunoprecipitation DNA samples were sent to quantification, sequence library construction, sequencing (Novogene, Beijing, China). The sequencing libraries were performed on the illumine HiSeq2000 system. Subsequent bioinformatic enrichment analysis was performed with clusterProfile. ChIP tracks were viewed in the Integrated Genome Viewer (IGV) version 2.3 on the mm10 genome build.

### Local delivery of infected MSCs to aged-mice

#### Transplantation of infected MSCs in cranial bone defects

For cells transplantation in aged mice, the mice were anesthetized by injection of Ketamine and Xylazine (100 mg/ml, 2:1, 1 ml/kg body weight). Then subcritical-sized cranial defects were generated at the flat surface of the cranium according to previous describe [46]. Briefly, after skin incision, two 1.0 mm cranial defects were created on both sides of the sagittal suture with a cylindrical low-speed carbide bur. Mice were blindly assigned into each gourps (*n* = 5–6 per group). Infected MSCs were trypsinized and diluted in phosphate-buffered saline (1 × 10^6^ MSCs in 10 µl PBS). Then, the cells were mixed with 10 mg β-TCP (Sigma-Aldrich) and transplanted into the bone defects. The periosteum and skin were closed with 5–0 monocryl sutures. The mice were sacrificed at six weeks following transplantation to perform micro-CT and histology analysis.

#### Infusion of infected MSCs in aged-mice femurs

For intra-femural BMSCs transplantation in aged mice, mice were blindly assigned into each gourps (*n* = 5–6 per group). After skin incision, infected MSCs (1 × 10^6^ MSCs in 20 µl PBS) were directly injected into the bone cavity at the distal condyles of femurs with 30-gauge needles according to previous described [24]. Right femurs were injected with Bmal1-overexpressed or miR-142-3p-inhibited BMSCs suspended in PBS, and the left femurs were injected with GFP-labeled or mCherry-labeled BMSCs. The ligament and skin were closed with 5–0 monocryl sutures. Femurs were harvested 8 weeks after surgery for analysis.

### μCT analyses

The harvested femurs and cranial bone tissues were fixed in 4% paraformaldehyde for 48 h and then stored in 70% ethanol at 4 °C before being processed. A μCT 50 micro-CT system (Scanco Medical, Bassersdorf, Switzerland) was used with a spatial resolution of 7 μm (70 kVp, 200 μA, 500 ms integration time). For the analysis of bone regeneration, the volume of interest was defined as a 1.5 mm cylindrical intra-femur area 250 μm distal to the growth plate. Bone volume fraction (BV/TV) was calculated within this region of interest (ROI). For the evaluation of trabecular bone regeneration, the trabecular number (Tb. N), trabecular thickness (Tb. Th) and trabecular separation (Tb. Sp) were calculated within the ROI.

### Histological and immunofluorescence analysis

Following micro-CT scanning, the femurs and cranium were decalcified in 10% EDTA (pH 7.4) and embedded in paraffin. Samples were cut into 5 μm thick sections with a microtome (Leica RM2235). Slices were stained with hematoxylin and eosin (H&E), Masson’s trichrome and Tartrate-resistant acid phosphatase (TRAP) staining according to the standard protocol (Sigma-Aldrich).

For immunostaining of the tissue sections, antigen retrieval was performed by boiling at 95 °C in 10 mM sodium citrate pH 6.0 for 20 min, and the slides were permeabilized in 0.25% Triton for 10 min prior to incubation in blocking solution (0.1% Tween 20, 10% goat serum, 1% BSA in PBS) for 20 mins, followed by incubation of the primary antibody with mouse anti-GFP (1:500, #NB600-597, Novusbio) or mouse anti-mCherrry (1:500, #NBP1-96752, Novusbio) and rabbit anti-ALP (1:100, #ab229126, Abcam) overnight at 4 °C. FITC conjugated goat anti-Rabbit IgG secondary antibody (1:100,#L30113, Sabbiotech) and rhodamine conjugated goat anti-mouse IgG secondary antibody(1:100,#L30314, Sabbiotech) were incubated for 1 h at RT. After mounting, the stained slides were photographed by Olympus FV3000 (Olympus, Japan).

For immunohistochemical analysis, tissue sections were deparaffinized, antigen retrieval prior to incubation in blocking solution (10% goat serum, 1% BSA in PBS) for 20 mins. The sections were incubated with rabbit anti-YAP (1:400, #1407 S, CST) or rabbit anti-p-YAP (1:1000, #13008, CST) primary antibodies at 4 °C overnight. The biotinylated secondary antibody was then incubated and visualized with 3,3’-diaminobenzidine (DAB). The percentage of positive-staining area and new bone formation area were measured with FIJI/ImageJ software.

### Statistical analysis

All data are presented as the mean ± s.e.m. In general, two-tailed Student’s *t-*test was used to evaluate the statistical significance between two groups, and one-way ANOVA followed by Turkey’s post hoc test was applied for multiple comparisons with GraphPad Prism 7 software. Two-tailed *p* values < 0.05 were considered statistically significant.

## Supplementary information

Table S1

Figure S1&2

Original Data File

## Data Availability

The datasets used and/or analyzed during the current study are available from the corresponding author on reasonable request.
