# Supplementary material for: Clock-modified mesenchymal stromal cells therapy rescues molecular circadian oscillation and age-related bone loss via miR142-3p/Bmal1/YAP signaling axis
Source: Cell Death Discov. 2022 Mar 12;8:111. doi: 10.1038/s41420-022-00908-7 (PMC8918353; doi:10.1038/s41420-022-00908-7)
Supplement: Supplementary file 1 — Table S1 [file 41420_2022_908_MOESM1_ESM.docx]

**Table S1. List of primers used for qRT-PCR in this study**.

| **Genes** | **Upstream (5’-3’)** | **Downstream (5’-3’)** |
| --- | --- | --- |
| *Gapdh* | ACCCAGAAGACTGTGGATGG | CACATTGGGGGTAGGAACAC |
| *Bmal1* | AACCTTCCCGCAGCTAACAG | AGAGGACCAGGGGACAT |
| *Clock* | AGGGCTGAAAGACGGCGAGAAC | GTCGGTGTGGAGGAAGGGTCTGA |
| *Per2* | AGAACGCGGATATGTTTGCTG | ATCTAAGCCGCTGCACACACT |
| *Rev-erbα* | GGATGCTTGCCGAGATGCAG | TCATAGAGAAGTCTTCCCAG |
| *p16* *^ink4a^* | CGTGAACATGTTGTTGAGGC | GCAGAAGAGCTGCTACGTGA |
| *P21^waf1/cip^* | CGGTGTCAGAGTCTAGGGGA | ATCACCAGGATTGGACATGG |
| *Alp* | AACCCAGACACAAGCATTCC | GCCTTTGAGGTTTTTGGTCA |
| *Runx2* | GGTACTTCGTCAGCATCCTATCAG | GCTTCCGTCAGCGTCAACAC |
| *Sp7* | ATGGCGTCCTCTCTGCTTG | TGAAAGGTCAGCGTATGGCTT |
