# Supplementary material for: Clock-modified mesenchymal stromal cells therapy rescues molecular circadian oscillation and age-related bone loss via miR142-3p/Bmal1/YAP signaling axis
Source: Cell Death Discov. 2022 Mar 12;8:111. doi: 10.1038/s41420-022-00908-7 (PMC8918353; doi:10.1038/s41420-022-00908-7)
Supplement: Supplementary file 2 — Figure S1&2 [file 41420_2022_908_MOESM2_ESM.pdf]

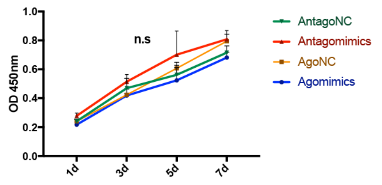


**Fig S1** CCK-8 assay at 2-day intervals after transfection of miR-142-3p agomimics and antagomimics. n.s, no significance.

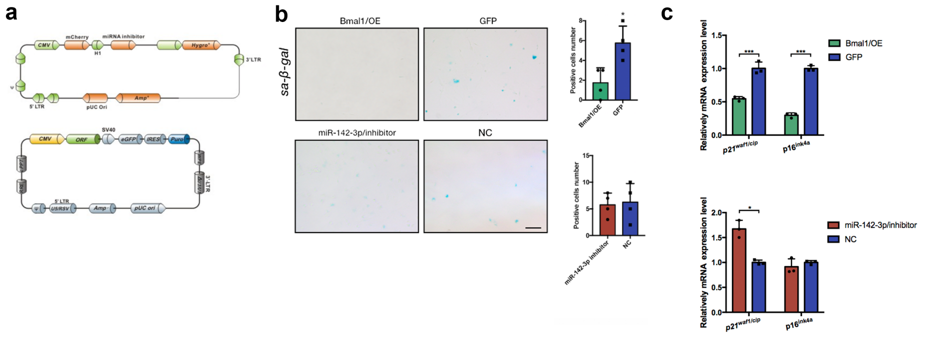


**Fig S2 a** Vector information about the Bmal1 overexpression and miR-142-3p inhibition plasmids. **b** SA-β-gal staining of Bmal1/OE, GFP, miR-142-3p/inhibitor and NC BMSCs, and quantitative analysis of the number of senescent cells. Scale bar: 200 μm *p < 0.05 by two-tailed Student's *t*-tests. **c** qRT-PCR analysis of the senescence-associated phenotype ($p16^{ink4a}$, $p21^{waf1/cip}$) in Bmal1/OE, GFP, miR-142-3p/inhibitor and miRNA/NC BMSCs. *p < 0.05, ***p < 0.001 by two-tailed Student's *t*-tests. Data are presented as mean ± SD.
