# Supplementary figures and images for: Clock-modified mesenchymal stromal cells therapy rescues molecular circadian oscillation and age-related bone loss via miR142-3p/Bmal1/YAP signaling axis
Source: Cell Death Discov. 2022 Mar 12;8:111. doi: 10.1038/s41420-022-00908-7 (PMC8918353; doi:10.1038/s41420-022-00908-7)

**Figure 1**

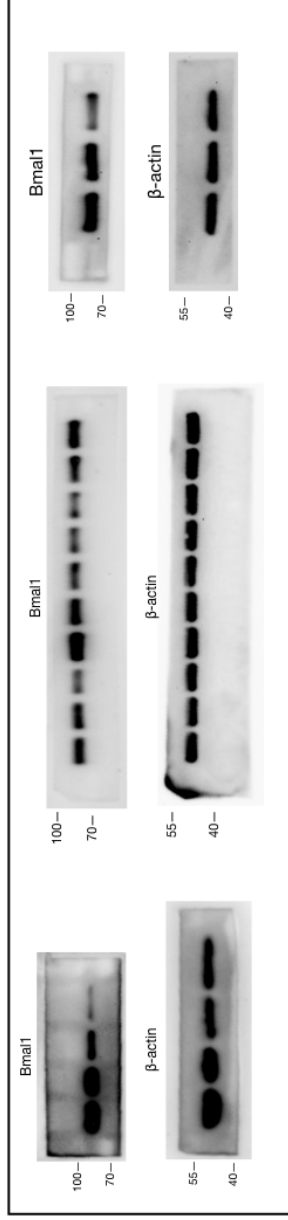

**Figure 2**

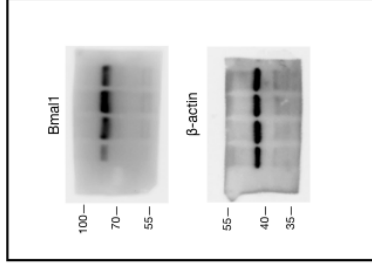

**Figure 3**

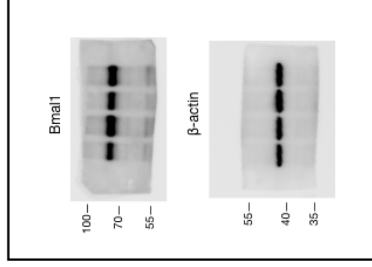

**Figure 4f**

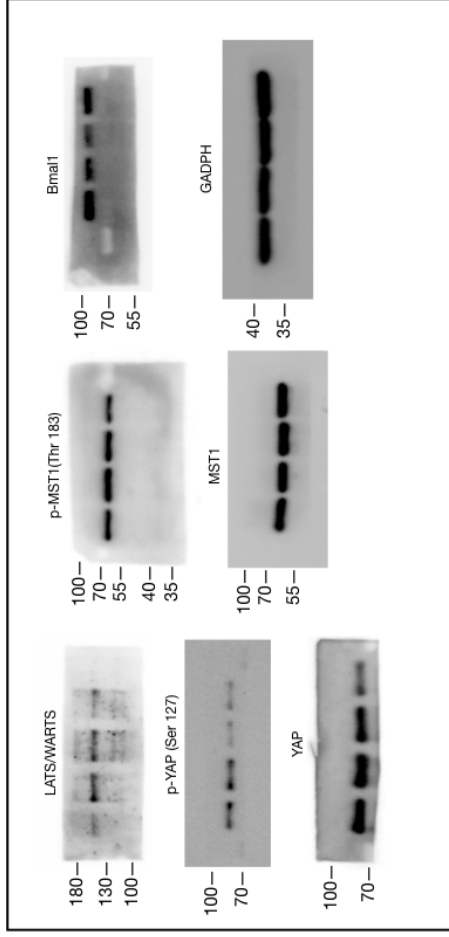

**Figure 4g**

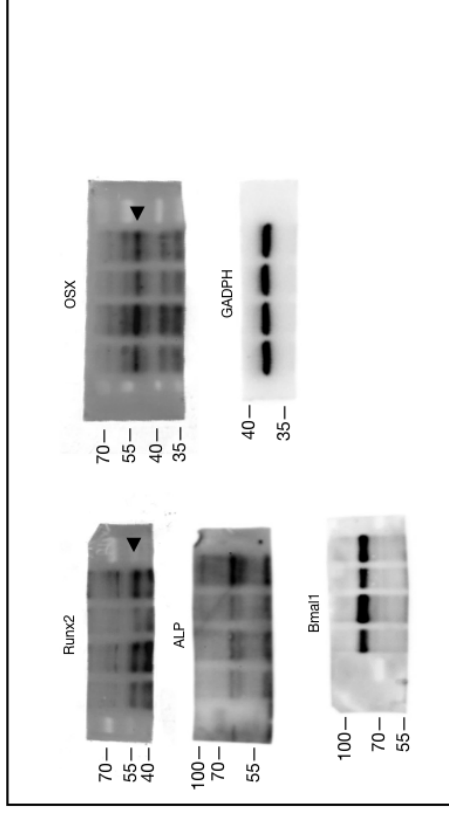

Supplement: Supplementary file 3 — Original Data File [file 41420_2022_908_MOESM3_ESM.pdf]
